# Supplementary material for: Reconciling Mining with the Conservation of Cave Biodiversity: A Quantitative Baseline to Help Establish Conservation Priorities
Source: PLoS One. 2016 Dec 20;11(12):e0168348. doi: 10.1371/journal.pone.0168348 (PMC5173368; doi:10.1371/journal.pone.0168348)
Supplement: S1 Dataset — (ZIP) [file pone.0168348.s002.zip › Taxa/Serra Norte/SN_2013/N4E/Lista N4E-71.pdf]

CAVIDADE N4E-0071

Classe	Ordem	Fam/Outros	Gên/Outros	Espécie	S-U	U-S	Zon.
Annelida	Oligochaeta			sp.1	3	14	E/A
Arachnida	Acari	(Oribatida)		sp.5		X	E
Arachnida	Acari	(Oribatida)		sp.9		X	E
Arachnida	Acari	Laelapidae		sp.5		X	E
Arachnida	Acari	Mesostigmata		jovem	X		E
Arachnida	Acari	Podocinidae	*Podocinum*	sp.1		X	E
Arachnida	Amblypygi	Charinidae	*Charinus*	sp.1		3	E/A
Arachnida	Amblypygi	Phrynidae	*Heterophrynus*	*longicornis*	2	4	A
Arachnida	Araneae	Drymusidae	*Drymusa*	*spelunca*	X	X	E
Arachnida	Araneae	Nesticidae	*Nesticus*	sp.1		X	A
Arachnida	Araneae	Ochyroceratidae		jovem	X	X	E/A
Arachnida	Araneae	Oonopidae		jovem		X	E
Arachnida	Araneae	Pholcidae	*Mesabolivar*	*aurantiacus*	X	X	E
Arachnida	Araneae	Pholcidae	*Mesabolivar*	sp.1		X	E
Arachnida	Araneae	Theridiidae	*Episinus*	sp.1		X	E
Arachnida	Araneae	Theridiosomatidae	*Plato*	sp.1	X	X	E/A
Arachnida	Opiliones	Escadabiidae		sp.2	X		A
Arachnida	Pseudoscorpiones	Bochicidae		jovem		X	A
Arachnida	Pseudoscorpiones	Chernetidae		sp.3	X		E
Chilopoda	Geophilomorpha	Geophilidae		sp.1		2	E
Chilopoda	Geophilomorpha	Schendylidae		sp.1		1	E
Chilopoda	Scolopendromorpha	Cryptopidae	*Cryptops*	jovem		1	E
Diplopoda	Polydesmida	Cyrtodesmidae		jovem	X		E
Diplopoda	Polydesmida	Fuhrmannodesmidae		sp.1	X	X	E/A
Diplopoda	Polydesmida	Paradoxosomatidae		sp.1		2	E
Diplopoda	Polydesmida	Pyrgodesmidae		sp.1	X	X	E/A
Diplopoda	Siphonophorida	Siphonophoridae		jovem		X	E
Entognatha	Collembola	Entomobryidae		sp.1	X		E
Entognatha	Collembola	Entomobryidae		sp.6	X		E
Entognatha	Collembola	Paronellidae		sp.1	X	X	E/A
Entognatha	Diplura	Campodeidae		sp.1	X	X	E
Insecta	Blattodea			jovem	1	1	E
Insecta	Coleoptera	Carabidae		sp.3	X		E
Insecta	Coleoptera	Scydmaenidae		sp.1		X	E
Insecta	Coleoptera	Staphylinidae	Pselaphinae	sp.30	X		E
Insecta	Coleoptera	Staphylinidae		sp.56	X		E
Insecta	Diptera	Chironomidae		sp.		X	E
Insecta	Diptera	Culicidae	*Culex*	sp.		X	E
Insecta	Diptera	Psychodidae	Phlebotominae	sp.	X	X	A
Insecta	Diptera	Sciaridae		sp.		X	E
Insecta	Diptera	Tipulidae	Tipulinae	sp.	X	X	E/A
Insecta	Heteroptera	Dipsocoroidea		sp.	X		E
Insecta	Heteroptera	Reduviidae	Emesinae	sp.8	1		E
Insecta	Heteroptera	Reduviidae	*Panstrongylus*	jovem	1		
Insecta	Homoptera	Cixiidae		jovem	X	X	E/A
Insecta	Hymenoptera	Formicidae	*Hypoponera*	sp.1		X	E
Insecta	Hymenoptera	Formicidae	*Hypoponera*	sp.2		X	E
Insecta	Hymenoptera	Formicidae	*Pachycondyla*	sp.2	X	X	E
Insecta	Hymenoptera	Formicidae	*Paratrechina*	sp.1	X	X	E/A

Insecta	Hymenoptera	Formicidae	*Pheidole*	sp.3		X	E/A
Insecta	Hymenoptera	Formicidae	*Solenopsis*	sp.1	X		E
Insecta	Isoptera	Termitidae	*Nasutitermes*	sp.	X		E
Insecta	Orthoptera	Phalangopsidae	*Paraclodes*	sp.	3		E
Insecta	Orthoptera	Phalangopsidae	*Phalangopsis*	sp.	12	4	E/A
Gastropoda	Pulmonata	Bulimulidae	*Naesiotus*	sp.		X	E/A
Gastropoda	Pulmonata	Systrophiidae	*Happia*	sp.	X		E
Malacostraca	Isopoda	Philosciidae		sp.1	X		E
Malacostraca	Isopoda	Philosciidae		sp.2		X	E
Malacostraca	Isopoda	Platyarthridae		sp.1		X	E
Malacostraca	Isopoda	Scleropactidae		sp.1	X		A
Nematoda				sp.1		X	E
Symphyla		Scutigerellidae		sp.	X	X	E/A
Amphibia	Anura	Strabomantidae	*Pristimantis*	cf. *fenestratus*	3		E
Mammalia	Chiroptera	Phyllostomidae	*Lonchophylla*	*thomasi*	5	2	E/A
